# La reforma a la salud pendiente en Chile: reflexiones en torno a una propuesta de transformación del sistema

**DOI:** 10.26633/RPSP.2017.170

**Published:** 2017-12-05

**Authors:** Camilo Cid, Andras Uthoff

**Affiliations:** 1 Organización Panamericana de la Salud/Organización Mundial de la Salud Washington DC Estados Unidos de América Organización Panamericana de la Salud/Organización Mundial de la Salud, Washington DC, Estados Unidos de América.; 2 Consultor senior independiente Santiago de Chile Chile Consultor senior independiente, Santiago de Chile, Chile.

**Keywords:** Reforma de la atención de salud, sistemas de salud, seguro de salud, seguridad social, Chile, Health care reform, health systems, insurance, health, social security, Chile, Reforma dos serviços de saúde, sistemas de saúde, seguro saúde, previdência social, Chile

## Abstract

Chile mantiene un sistema de salud segmentado por riesgos e ingresos. Una Comisión Presidencial encargada en 2014 planteó dos escenarios para el sistema de salud según el horizonte de tiempo en que se esperaban sus resultados. Para el largo plazo propuso una visión hacia dónde debiera dirigirse el sistema de salud chileno, conviniendo en un seguro público único. En lo inmediato, propuso una transición que consistía en regular a las instituciones de salud previsional para que funcionaran bajo reglas y principios de seguridad social. Los planteamientos centrales se sustentaron en evidencia internacional de sistemas que han alcanzado éxito en ofrecer acceso y cobertura universal de salud a sus habitantes, mediante las transformaciones y regulaciones apropiadas. El análisis realizado por la Comisión implicó la inauguración de un nuevo paradigma para las políticas de salud en Chile. Uno que señala que la estrategia utilizada hasta ese momento, de promover mayor competencia y libertad de elección en mercados de seguros de salud, no ha dado resultados, y que para avanzar hacia el acceso equitativo a la salud es necesario poner en el centro el derecho a la salud y los principios de solidaridad y equidad, así como valorar el avance en el mundo, reconociendo que el esquema chileno se aleja de las mejores prácticas en cuanto a diseño de los sistemas de salud. La propuesta no se ha implementado aún, y será necesario plantear una implementación acelerada de la visión de largo plazo. La experiencia es relevante para otros países de la Región de las Américas que discuten los problemas de la segmentación en salud.

## Abstract (en)

Chile maintains a health system segmented by risks and income. A Presidential Commission convened in 2014 presented two scenarios for its health system in accordance with a timeline of expected results. For the long term, it proposed a vision for the direction of the Chilean health system that would converge in a single public insurance. For the short term, it proposed a transition that consisted of regulating pension health institutions to function under social security rules and principles. The central approaches were based on international evidence of systems that have achieved success in offering universal health access and coverage to the population, through appropriate transformations and regulations. The analysis carried out by the Commission signaled the beginning of a new paradigm for health policies in Chile. This points out that the strategy used previously—to promote greater competition and freedom of choice in health insurance markets—has not yielded results, and that in order to advance towards equitable access to health it is necessary to focus on the right to health and the principles of solidarity and equity, as well as assessing progress in the world, recognizing that the Chilean scheme moves away from the best practices in terms of the design of health systems. The proposal has not been implemented yet, and it will be necessary to propose an accelerated implementation plan for its longterm vision. Chile’s experience is relevant for other countries in the Region of the Americas that discuss the problems of health segmentation.

## RESUMO

O Chile mantém um sistema de saúde segmentado por riscos e renda. Uma Comissão Presidencial delegada em 2014 propôs dois cenários para o sistema de saúde de acordo com o horizonte temporal em que seus resultados eram esperados. Para o longo prazo, foi indicadauma visão de onde o sistema de saúde chileno deveria chegar, concordando com um único seguro de saúde público. De forma imediata, foi recomendada uma transição que consiste em regular as instituições de seguro de saúde para funcionar de acordo com as regras e princípios de seguridade social. As abordagens centrais foram baseadas em evidências internacionais de sistemas que alcançaram sucesso em oferecer acesso e cobertura de saúde universal aos seus habitantes, através de transformações e regulamentos adequados. A análise realizada pela Comissão envolveu a inauguração de um novo paradigma para as políticas de saúde no Chile. Uma sinalização foi sobre a estratégia utilizada até esse momento, de promover uma maior concorrência e liberdade de escolha nos mercados de seguros de saúde, não tem produzido resultados, e que, para avançar ao acesso equitativo à saúde, é necessário colocar no centro da estratégia o direito à saúde e os princípios de solidariedade e equidade, bem como avaliar o progresso no mundo, reconhecendo que o esquema chileno se afasta das melhores práticas em termos de projetar sistemas de saúde. A proposta não foi implementada até o momento, e será necessário propor uma implementação acelerada da visão de longo prazo. A experiência é relevante para outros países da Região das Américas que discutem os problemas de segmentação da saúde.

A pesar de los positivos resultados generales en salud en el marco regional, Chile mantiene un sistema de sanitario segmentado por riesgos e ingresos, poco eficiente, que destaca por sus niveles de inequidad.

Las personas de mayores ingresos pueden salir del *pool* solidario de toda la sociedad, representado por el Fondo Nacional de Salud (FONASA) y llevar la cotización de seguridad social a fondos privados separados y estancos, que basan su relación contractual en el riesgo. El segmento privado, las Instituciones de Salud Previsional (ISAPRE) con sólo el 18,5% de la población, ocupa cerca de la mitad de los recursos que se gastan en salud en el país, que representa cerca del 8% del producto interior bruto (PIB), a través de gastos en primas y pagos directos de los hogares que constituyen un tercio del total.

El sistema se financia con una prima de seguridad social obligatoria del 7% de las remuneraciones (con tope de US$ 3 000 anuales) aportada sólo por los trabajadores; a esto se adiciona un aporte fiscal para FONASA, que supone el financiamiento de aquellas personas que no pueden cotizar. En las ISAPRE, a la prima del 7%, se adiciona una prima fija para financiar las garantías explícitas de salud (GES) y una prima adicional que compensa cualquier diferencia de la suma anterior con el riesgo esperado en cada contrato, de tal forma que las primas resultan ser muy variables y si se compara con la renta, la cotización media resulta ser más del 10%.

En 2010, el Tribunal Constitucional (TC) resolvió eliminar de la ley las facultades que permitían explícitamente a las ISAPRE ajustar por sexo y edad las primas al vender sus planes, aduciendo que diferenciar de ese modo entre personas era discriminatorio y, por tanto, inconstitucional. Las ISAPRE se ajustaron a este nuevo escenario continuando las prácticas de discriminación de riesgos, y siguieron elevando los precios de forma unilateral. Pero el debate se abrió transformándose en un tema que reiteradamente ocupa la agenda pública.

En marzo de 2014, la presidenta Michelle Bachelet nominó una Comisión Asesora Presidencial (en adelante, la Comisión) para que la apoyara con *“propuestas de cambios y reformas para que el acceso a la salud pueda ejercerse como un derecho de la seguridad social, sin importar a qué régimen estén afiliadas las personas”* (1). La Comisión la conformaron 18 expertos; la mayoría de ellos formuló la posición que aquí se discute.

En este trabajo se discute la propuesta, constatando su acierto en la nueva manera de plantear la reflexión estructural acerca del sistema de salud chileno y se agregan nuevos elementos que la apoyan. Se desarrollan tres reflexiones: la primera asociada al diagnóstico del sistema de salud; la segunda se refiere a la propuesta propiamente como tal, a su coherencia con la necesidad de cambios estructurales urgentes; y la tercera da cuenta de algunas visiones contrarias a los principios de acceso universal a la salud y de seguridad social como la solidaridad y la equidad, que aparecieron durante la discusión.

## LA SITUACIÓN DEL ASEGURAMIENTO DEL SISTEMA DE SALUD CHILENO

Desde la introducción de la opción de cotizar en un mercado de seguros privados de salud a inicios de los ochenta, el aseguramiento de salud en Chile se caracteriza por la discriminación, la falta de transparencia, copagos excesivos, alzas unilaterales de los precios de las primas, etc. Ninguno de los preceptos de seguridad social se cumple: no existe solidaridad ni equidad, y tampoco eficiencia ni sostenibilidad. El sistema ISAPRE ha estado implícitamente soportado por el sistema público que asume a los desplazados justamente cuando estos ofrecen mayores posibilidades de gasto, ya que en general, las personas deben dejar el sistema ISAPRE cuando adquieren una enfermedad o envejecen *(*2). De hecho, el sistema ISAPRE presenta una baja proporción de adultos mayores (sólo 5,6%, el 94,4% están en FONASA), con un índice de envejecimiento de 35 frente a 82 de FONASA, con predominio de hombres (116 hombres por cada 100 mujeres) y una alta proporción de población económicamente activa (71% contra 63% de FONASA) (2).

Los problemas del esquema de seguro chileno han sido descritos en detalle por varios autores (3-9), y fueron tratados por la Comisión que agregó antecedentes importantes, como aquellos asociados a la desigualdad y discriminación existente al interior del sistema privado, y la constatación que la población adscrita al FONASA presenta consistentemente peores estados de salud y utilización, con una mayor prevalencia de daño debido a la particpación del envejecimiento, que ha sido corroborada posteriormente por otros trabajos (2,10).

El diagnóstico técnico indica que en un mercado de seguros individuales de salud como el de las ISAPRE, las aseguradoras cobrarán una prima suficientemente alta como para cubrir los costos esperados de cada contrato, los gastos de administración y las utilidades, esto es, de acuerdo al principio de equivalencia. Dada la fuerte variación en los gastos de salud entre individuos, esta estrategia hará que, con certeza, la mayoría de las personas de alto riesgo no puedan pagar su seguro o lo hará con un gran impacto en sus ingresos (11, 12).

De este modo, las ISAPRE, al identificar los costos esperados mayores, desplazan o cobran caro por niños pequeños, mujeres en edad fértil, adultos mayores y personas con enfermedades crónicas o preexistentes. Estos cobros, además, van subiendo año tras año; de hecho, la diferencia de prima entre la población joven y la población anciana supera las 3 veces, y la de la mujer en edad fértil más que duplica a la de los hombres. Los copagos, que en promedio ya son altos (35% del costo total), son superiores para las mujeres a cualquier edad (2). Por otra parte, la ausencia de regulación permite el constante traspaso de costos a los beneficiarios mediante alzas periódicas de las primas de los planes en uso.

La falta de transparencia es otro aspecto importante; el proceso de selección y los criterios de equivalencia prima–riesgos, provocan que existan más de 65 000 planes en diversas modalidades de acceso y cobertura financiera que impiden cualquier decisión informada de las personas. Estos planes aumentan en un millar anualmente, producto de que los precios de venta de nuevos planes son libres y las aseguradoras los ajustan conforme a sus expectativas.

Para la Comisión el sistema de salud chileno es segmentado, con una industria de aseguramiento privado sustituto primario; caracterizado por un mercado con integración vertical, donde el principio de libertad de elección, sobre el cual descansa, no se cumple dado que al menos el 40% de los afiliados están cautivos. En otras palabras, estos no pueden cambiarse de ISAPRE debido a que ninguna otra los recibirá por presentar preexistencias (o edad inconveniente) y deben permanecer en una ISAPRE que intenta, además, desprenderse de ellos (2); donde FONASA cubre solidariamente a más del 75% de la población sin que el sistema considere un *pool* social para eliminar la discriminación por ingresos y condiciones de salud o compensar al FONASA por los desplazados desde las ISAPRE.

La Comisión modificaba la forma de analizar los problemas estructurales de salud en las decisiones de política. En efecto, anteriormente las reformas no se enfocaron en la segmentación y más bien reforzaron criterios mercantiles sin considerar la lógica de seguridad social y sus principios. Los siguientes son tres ejemplos de aquello: a) con la reforma de GES de 2005 se reconoció el uso de factores diferenciales basados en el sexo y la edad para aplicar a las primas legalizando la diferenciación de riesgos; b) aun cuando los precios de las primas han estado siempre ajustados con la inflación y que en 2005 se intentó regular el alza real, esta se sigue produciendo a voluntad del seguro, sin intervención de la otra parte del contrato; c) la creación de las GES autorizó a las ISAPRE el cobro de una prima de libre fijación que forma parte de la prima total, que considerada de manera independiente subsidia ganancias y gastos de administración debido a que el bajo gasto en GES no guarda relación con dicha prima .

**FIGURA 1.**
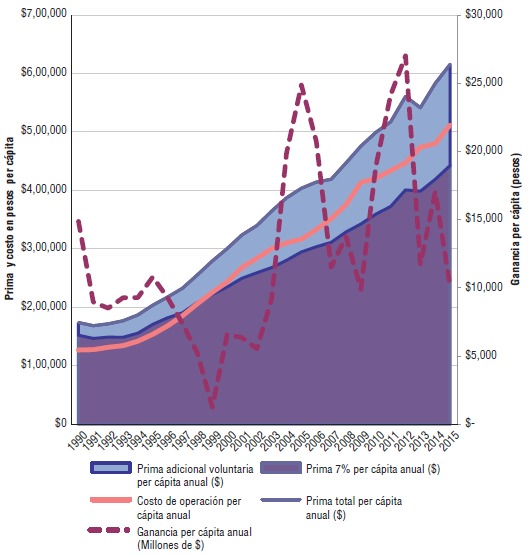
Evolución de los precios reales de las primas, los costos y ganancias de ISAPRE abiertas, 1990-2015 (primas en pesos per cápita de 2011 y ganancias en millones de pesos de 2011)

Una serie de largo plazo permite establecer (Figura 1) que la tasa de crecimiento de los precios reales de las primas totales per cápita ha sido de 5,2% anual y que el precio total medio de las primas per cápita de 2015 resulta ser 3,5 veces más elevado que en 1990. La prima adicional voluntaria per cápita (aquella parte que está por encima del 7%) pasó de ser un cuarto del total en 1990, a representar casi un tercio en 2015, siendo la de 2015 ocho veces mayor a la de 1990, ya que creció a un promedio anual real de 8,9%. La prima de seguridad social del 7% per cápita, que crece con los salarios, también aumentó y lo hizo a una tasa anual promedio un tanto menor que la de los gastos. El control de los costos, que es la principal función de un seguro de salud, no ocurre y estos suben a la par que estos ingresos (Figura 1).

***Fuente:*** Elaboración propia con datos de la Superintendencia de Salud.

Nota: A la serie 1990–2012 publicada por la Superintendencia se ha agregado el periodo 2013-15 con cálculos propios de los autores, a partir de los datos anuales publicados por esta Institución.

**FIGURA 2.**
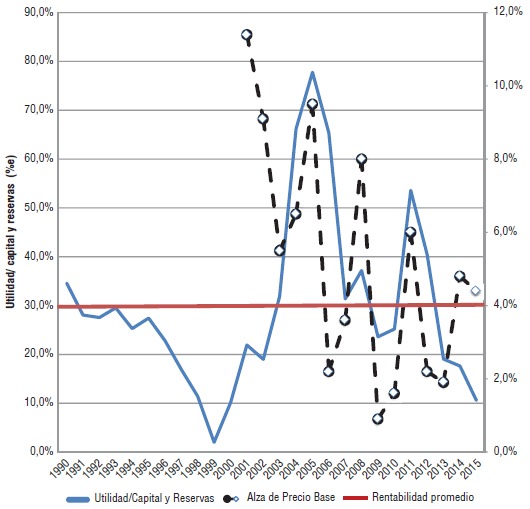
Rentabilidad del sistema ISAPRE 1990-2015 y alza del precio base 2000-2015

El crecimiento de las primas ha permitido al sistema privado mantener altas ganancias y una tasa de rentabilidad media de 30% al año, que nunca ha sido negativa, con fluctuaciones que la han llevado desde un breve período por debajo del 10%, hasta períodos de rentabilidad superior al 30%, con un máximo de 78% en 2008 (Figura 2).

## LA PROPUESTA Y SU CARÁCTER ESTRUCTURAL: URGENCIA Y TRANSFORMACIÓN

La Comisión tuvo en cuenta la experiencia internacional que muestra que es posible responder a los desafíos que impone el funcionamiento del sistema privado por la vía de la regulación (11–13) y mediante un diseño estratégico, en que las medidas inmediatas sean parte de un tránsito hacia un futuro deseado en cuanto a acceso y cobertura, lo que implica la transformación del sistema (14,15).

De esta forma, se debatió sobre un conjunto de medidas regulatorias destinadas a garantizar el acceso a las prestaciones de salud, en forma independiente de los niveles de riesgos, a la brevedad posible. Para ello se propuso terminar con los problemas de discriminación y selección de asegurados; ello implicaba necesariamente medidas para controlar las alzas de los precios de las primas, generar un conjunto amplio y único de beneficios estandarizados en cuanto a prestaciones y copagos, considerando que las grandes diferencias en los planes privados se dan en la cobertura financiera (2), junto con considerar una mirada global del financiamiento que considerara a la salud como un derecho y que definiera un camino para transitar hacia un nuevo sistema de salud en un plazo mayor.

La Comisión adhirió a principios de la seguridad social, como la solidaridad, la universalidad y la sostenibilidad. Un tema central para el cumplimiento del mandato era la definición del rol de las ISAPRE en ese contexto normativo. Al respecto, se acordó que *“Las ISAPRE sí son parte de la seguridad social, lo que está asociado a la obligatoriedad de la cotización del 7% de la renta para Salud…”*. (2). En contraste, las ISAPRE se han comportado históricamente como seguro de carácter voluntario y sustituto y sus representantes las conciben como una alternativa ofrecida por empresas privadas en libre competencia. Con esa resolución, en cambio, la Comisión estimó que el hecho de que ellas administren el 7% que los trabajadores aportan obligatoriamente a la seguridad social era categórico, lo que constituye otro acierto por cuanto ello es coherente con las clasificaciones internacionales de los seguros de salud. El Cuadro 1 muestra la gama posible de esquemas de seguros de salud y una forma de entender el aseguramiento privado; este es principalmente voluntario y se desarrolla por fuera de la cobertura social o pública de los países, de forma complementaria y/o suplementaria a ella.

La mayoría de los miembros de la Comisión consideraron los recursos provenientes de las cotizaciones de seguridad social idóneos para ser utilizados en el financiamiento de un acceso igualitario y solidario entre personas de diferentes riesgos sanitarios, en un contexto en que las aseguradoras son responsables financieramente de todo el ciclo vital de sus afiliados. Para ello se optó por combinar dos vías complementarias con medidas de corto y largo plazo.

***Fuente:*** Elaboración propia con datos de la Superintendencia de Salud.

### El corto plazo: regulación y solidaridad

Para el corto plazo se propuso regular el precio de las primas de manera directa e indirecta, eliminar las preexistencias y permitir la afiliación abierta (libre elección de asegurador) para acceder a un conjunto amplio de beneficios único financiado con el 7%, con cobertura financiera estandarizada. Esto es, un sistema de copagos conocidos y menores, que incluye un gasto anual tope respecto de la renta para evitar el empobrecimiento por eventos de salud.

En materia de solidaridad se propuso aplicar de inmediato un fondo de compensación de riesgos entre ISAPRE conformado con el conjunto del 7% obligatorio de seguridad social que financiaría el conjunto de beneficios universal.

También se propuso la creación de un fondo mancomunado universal en el conjunto del aseguramiento, es decir, entre FONASA y las ISAPRE, para financiar prestaciones universales priorizadas, por primera vez de manera completamente solidaria en el país. Este sería el inicio del fondo único, que se iría ampliando con el tiempo junto con la puesta en práctica de otros elementos. Se buscaba mancomunar el máximo posible los fondos de la seguridad social de salud, pasando de una segmentación de 8 fondos existentes (FONASA + 7 ISAPRE abiertas) y miles de planes, a sólo dos fondos (FONASA y uno de las ISAPRE), un solo conjunto amplio de beneficios y el fondo mancomunado creciente entre ambos subsistemas.

Finalmente, en el corto plazo se reconocía la posibilidad de existencia de una prima comunitaria (igual para todos los miembros) adicional al 7% por ISAPRE, regulada, que reconocería las preferencias de las personas que demanden cobertura en clínicas privadas durante la transición, exigiendo también condiciones de trabajo en red por parte de los proveedores.

Este conjunto de medidas encuentran su apoyo en los marcos regulatorios y la experiencia documentada de varios países europeos con problemas de selección de riesgos como Alemania, Bélgica, Holanda, Hungría, Israel, la República Checa, Suiza (11–13,16) e incluso Estados Unidos, donde en 6 años se promovió la incorporación de importantes grupos de personas al aseguramiento (seguro obligatorio) junto con la regulación de primas comunitarias, afiliación abierta para impedir la discriminación y una expansión importante de los programas públicos (17).

En otras materias se proponía separar claramente el aseguramiento complementario y suplementario privado, de la seguridad social. Esta sería una regulación necesaria que, en el futuro, alojaría además a las propias ISAPRE o sus herederas cuado se retirasen de la seguridad social.

Se propuso fortalecer el sistema público de salud durante la transición para prepararlo para la integración del sistema único. Se cuestionó la integración vertical, reafirmando el concepto de exclusividad de giro de aseguramiento de las ISAPRE que la ley exige^3^ y se propuso implementar sistemas de pago mixtos, eficientes en la relación entre pagadores y proveedores para evitar el traspaso de riesgo directo hacia los beneficiarios y viabilizar la regulación de precios. Este conjunto de medidas de corto plazo permitiría perfilar el sistema hacia un cambio mayor.

### El largo plazo: el carácter estructural de la propuesta

En consideración al mandato de dotar al sistema privado de salud de características reales de seguro social (1), la Comisión coincidió en que, en un escenario de largo plazo, la existencia de una sola entidad pública que mancomunase todo el financiamiento disponible y lo destinase al acceso y cobertura universal para todos los habitantes resolvería el problema de falta de acceso e inequidad en el conjunto del sistema de salud chileno. De esta forma, la propuesta de corto plazo resultaba coherente con una mirada más profunda del futuro y demandaba tomar conciencia de la necesidad de una reforma más amplia, con plazos e hitos definidos. La Comisión considero que esa mirada, la de definir claramente el horizonte, era necesaria para evaluar aquellas medidas que, planteadas para el plazo inmediato, fueran también conducentes a lograr el objetivo de la propuesta.

**CUADRO 1.** Seguridad social de salud y tipos de aseguramiento voluntario privado

Caracter del seguro	Tipo deaseguramiento	Definicion	Principiopredominante	Naturaleza de las Primas	Ejemplo de paises
Publico obligatorio	Seguro de seguridad social de salud	Aseguramiento social amplio en cuanto a prestaciones y cobertura financiera	Principio de solidaridad y derecho a la salud	Primas solidarias obligatorias proporcionales al salario compartida por empleados y empleadores	Alemania, Holanda, Belgica
Privado voluntario	Seguro primario principal	Entregan cobertura de salud porque no hay cobertura social o las personas no son elegibles para ella	Principio de equivalencia	Precios de mercado asociados a riesgos	Estados Unidos (Medicare y Medicaid)
	Seguro primario sustituto	Las personas pueden tener derecho a la cobertura social en prestaciones y financiamiento, pero han optado por sustituirla por seguros privados.	Principio de equivalencia	Precios de mercado asociados a riesgos	Seguro privado de Alemania (10% de la poblacion)
	Seguro duplicado	Ofrecen cobertura ya incluida en el seguro social o servicio nacional de salud (para acceso a proveedores diferentes, por ejemplo). No eximen a las personas de la contribucion al seguro social	Principio de equivalencia	Precios de mercado en algunos casos regulados asociados a riesgos	Brasil (Salud suplementar), Australia
	Seguro complementario	Mejoran la cobertura financiera del seguro social o servicio nacional de salud reduciendo el gasto de bolsillo o reembolsando por prestaciones no totalmente cubiertas por el seguro basico o establecidas en el contrato	Principio de equivalencia	Precios de mercado asociados a riesgos.	Seguros complementarios en Francia, Suiza, Corea del Sur
	Seguro suplementario	Agregan prestaciones adicionales y que no estan incluidos en los planes de beneficios de la seguridad social o servicio nacional de salud. Suplementan con prestaciones el aseguramiento social	Principio de equivalencia	Precios de mercado, a veces regulado, asociados a riesgos	Seguros Suplementarios en Sudafrica, Irlanda, Holanda

***Fuente:*** Elaboración propia en base a referencias.

Este planteamiento se ve reforzado con las evidencias que muestran que los países pueden transitar hacia el cambio profundo de sus sistemas de salud. A los casos conocidos de los años ochenta de Brasil y España^4^, pueden agregarse otros como son los de Corea de Sur y Taiwán (14,15) durante los 90; Estonia y Polonia (16) en esos mismos años; Turquía de manera más reciente a partir de 2003 (18); Costa Rica en los noventa^5^ y Uruguay hace sólo unos años en 2008 (19), en América Latina.

En todos estos casos se pasó de un sistema con aseguramiento múltiple y segmentado a uno único en un proceso planificado de cambios de aproximadamente una década de duración, para construir un sistema de aseguramiento y fondo único de salud con un solo pagador, bajo los principios de seguridad social de acceso y cobertura universal. En todos los casos, los éxitos mostrados son reconocidos internacionalmente, de tal forma que junto con el aumento del gasto público se produce una disminución en los gastos directos de los hogares y una mejoría sustantiva en los indicadores de salud (14,15,18,19).

El cuadro 2 destaca los casos de algunos países según cuatro variables claves: el tránsito del esquema de aseguramiento, y la regulación de los precios, los beneficios y los pagos a los proveedores. También se incluyen datos acerca del aseguramiento privado voluntario que van en la línea de lo señalado por la Comisión.

Finalmente, en 2014, Chile (junto al resto de los países de la Región de las Américas) adhirió a la propuesta estratégica de acceso y cobertura universal de salud de la Organización Panamericana de la Salud que, bajo los principios de derecho a la salud, equidad y solidaridad, y en un marco de eficiencia, propone el aumento del gasto público y la eliminación del gasto de bolsillo, y promueve el uso de fondos mancomunados que terminen con la segmentación (20). La propuesta de la Comisión se encuentra en línea con estos argumentos.

## VISIONES CONTRARIAS A PRINCIPIOS DE UNIVERSALIDAD EN SALUD

Una minoría de la Comisión planteó la posibilidad de avanzar hacia un sistema de multiseguros como mirada de largo plazo del sistema de salud, con la conformación de un fondo con participación de las ISAPRE y FONASA (2). Otra posición que finalmente adhirió a la posición minoritaria no compartía el diagnóstico y propiciaba que no era necesario integrar a las ISAPRE en la lógica de la seguridad social, que el sistema no presentaba un problema estructural que solucionar y que estaba legítimamente destinado a cubrir a aquellos de mayores ingresos de la población. Estos miembros de la Comisión postulaban que con pequeños cambios que limitaran la judicialización —que genera gastos legales a las aseguradoras ya que deben cubrir los costos de aproximadamente 150 mil juicios perdidos por año—, las ISAPRE podrían seguir funcionando^6^.

**CUADRO 2.** Características principales en casos de transformaciones en el aseguramiento de la salud (países escogidos)

País	Principios, preponderantes y esquema de aseguramiento	Regulación de los ingresos al seguro	Regulación de los beneficios	Regulación para el funcionamiento de la asignación de recursos	Regulación seguros voluntarios compl./suplem.
Corea del Sur	Transitó de multiseguros (más de 300) a seguro público único a partir de 1999, bajo principios de eficiencia y solidaridad	Prima solidaria proporcional al ingreso compartida en partes iguales entre empleador y empleado de 4,5%	Conjunto amplio de beneficios. Con coseguro importante (20% hospitalario y entre 35 y 50% en lo ambulatorio)	Un solo pool de riesgos y pagador único. Los pagos se realizan bajo distintas modalidades, como fee for service y GRD (hospitales) y tarifas en lo ambulatorio	Suplementario, pero con poca participación en el gasto total (menos del 5%)
Estonia	Transitó de multiseguro (22) a seguro público único a partir de 1994 y 2001	Contribuciones del 13% (empleadores) que constituyen cerca del 65% del financiamiento, el resto de impuestos. 95% de la población está cubierta con 45% de no contribuyentes	Conjunto amplio de beneficios definido por exclusión o lista negativa. Existen copagos estandarizados y conocidos.	2% del pool es retenido para enfermedades de alto costo o raras La primera etapa de la asignación de recursos ocurre capitadamente desde el Fondo a las 4 regiones de salud existentes. 50% del financiamiento hospitalario se asigna utilizando los GRD	Mercado suplementario muy pequeño de solo unos cientos de personas (menos del 0,01%) no elegibles para la cobertura del financiamiento público (Fondo del seguro de salud de Estonia)
Polonia	Transitó de multiseguro a seguro público único en 2003	Contribuciones de seguridad social del 9% de la nómina	Conjunto amplio de beneficios definido por exclusión o lista negativa, con algunos servicios positivamente listados (procedimientos dentales, medicamentos, entre otros)	Primer nivel pagado per cápita ajustado por edad. En hospitales pagos basado en caso de acuerdo a departamento, estadía y complejidad del hospital y diagnósticos.	Existe aseguramiento suplementario privado con el 3,1 a 3,9% de la población, pero con sólo el 0,6% del gasto en salud
Turquía	Transitó desde multiseguro a seguro público único bajo principios de equidad, eficiencia, efectividad y responsabilidad	Contribuciones de seguridad social y 75% ingresos desde impuestos generales	Conjunto de beneficios ampliado de manera incremental	rograma de transformación de salud en 10 años. Distribución de presupuesto total en base a servicios provistos. Importancia de la atención primaria.	Seguros privados complementario y suplementario pero tienen una participación moderada (4,4% del gasto total en salud)
Uruguay	Transitó de multiseguro a seguro público único con proveedores administrando riesgos a partir de 2008, bajo principio de solidaridad	Prima solidaria proporcional al ingreso, escalonada	Conjunto amplio de beneficios definido por exclusión y equivalente en costos a los ingresos de la seguridad social	Un pool de riesgos que se reparte mediante ajuste de riesgos solidario con la totalidad del financiamiento de la seguridad social a los proveedores organizados en mutuales y sector público.	No pueden ser ofertados por el aseguramiento social. No pueden ofrecer coberturas primarias

***Fuente:*** Elaboración propia en base a referencias.

GRD: grupos relacionados de diagnóstico.

La posición de mayoría fue que no era necesario —y era más bien incierto e ineficiente— mantener la alternativa de multiplicidad de aseguradoras, más allá de la transición de corto plazo, lo que exigía contar con un período de evolución similar a los de las experiencias estudiadas y con una regulación adecuada de los seguros privados a cambio de continuar utilizando el 7% de la seguridad social sólo durante la transición (2).

La defensa de las ISAPRE y las críticas aparecidas posteriormente a la presentación del informe apuntaron a elementos como los siguientes: a) no aceptar el concepto de prima de seguridad social en las ISAPRE sino, por el contrario, mantener el concepto de propiedad privada que se expresa en precios de planes individuales. Esto constituye una manera de ignorar el carácter social del financiamiento obligatorio y significa conservar la filosofía actual de la industria privada; b) no aceptar la idea de mancomunar los recursos de la seguridad social reflejados en la contribución del 7% y a cambio formar un fondo ISAPRE con un aporte igual a una tarifa plana de un plan mínimo no solidario y básico. Este tipo de perspectiva se observa en algunos casos en el mundo de los seguros privados voluntarios (16,21-24), en que estos seguros son un mercado secundario que aplican coberturas suplementarias de prestaciones no cubiertas por el seguro público o social primario y no forman parte ni de la seguridad social, ni de un seguro social o un servicio nacional de salud. Generalmente tienen una participación minoritaria en el financiamiento (23) (Cuadros 1 y 2); c) que el cambio propuesto podía provocar exceso de movilidad de los beneficiarios de manera que se cuestionase la sostenibilidad del sistema de salud en su conjunto.

Se han hecho estimaciones que negaron este argumento (2). Sin embargo, la forma de reducir esta incertidumbre de manera radical es asumiendo el compromiso de largo plazo y la certeza de que se avanza hacia un solo sistema de salud para toda la sociedad.

### Comentarios finales

La Comisión Asesora Presidencial de Chile del año 2014 cambió el marco de reflexión que venía ocurriendo en las políticas estructurales del sistema de salud. El cambio de paradigma que propuso se basa en la idea de que la incursión del mercado sin regulación, colocando el énfasis solo en promover mayor competencia, ha terminado en exclusión y sin garantizar los derechos de acceder a prestaciones de salud. Chile necesita asumir que su sistema de salud se encuentra alejado de parámetros razonables y avanzar a una reforma que termine con la segmentación y abra paso a una nueva generación de transformaciones que eleve los niveles de equidad y acabe con el derroche de recursos impuesto por la ineficiencia social del sistema estanco.

Resulta urgente la implementación del modelo propuesto por la Comisión con una transición más acelerada. Se debería avanzar lo más rápido posible en mancomunar todos los fondos disponibles en uno solo, dando paso a un ente público pagador único sin intermediación de seguros y sin más mediación que las coordinaciones y regulaciones de redes públicas establecidas (a las cuales podrán adherir proveedores privados). Los problemas de eficiencia y equidad, se resolverán entonces en las formas de asignación de los recursos. Estas deberán promover la integración de las redes y un modelo centrado en la atención primaria, con acceso equitativo y universal. La experiencia de países que han transitado ese camino será vital en este tránsito; en todo caso, se habrá dado un paso definitivo en la solidaridad necesaria en el sistema de salud chileno.

Esta experiencia es también importante para varios países de la Región que tienen sistemas segmentados de salud y puede contribuir a la reflexión acerca de sus posibilidades de avance.

### Declaración.

Las opiniones expresadas en este manuscrito son responsabilidad de los autores y no reflejan necesariamente los criterios ni la política de la RPSP/PAJPH o de la OPS.

## Footnotes

3 Sin embargo, en los hechos la forma de conformación de las sociedades privadas y los grupos económicos permiten evadir el espíritu de esta regla.

4 España pasó de un sistema de seguridad social de salud tipo Bismark a un Servicio Nacional de Salud desde fines de los 70 y por un período de aproximado de 10 años. Brasil en 1988 generó el Sistema Único de Salud en el marco del retorno a la democracia, que se desarrolló rápida y exitosamente.

5 Desde su creación en 1941 la Caja Costarricense de Seguridad Social presenta grandes hitos. En los 60 se decidió la universalización del seguro a toda la población y en los 70 se unificó la gestión de los hospitales. Sin embargo, en los 90 se produjo una reforma que consideró el fortalecimiento del rol rector del Ministerio de Salud y se confirmó la función de pagador y seguro único de la Caja, a diferencia de lo que se estaba promoviendo en la Región.

6 Ello se puede apreciar en la lectura del informe de la Comisión a través de las abundantes notas a pie de página donde se consignaron los desacuerdos de este grupo de comisionados.
